# Loss of CENP-F Results in Dilated Cardiomyopathy with Severe Disruption of Cardiac Myocyte Architecture

**DOI:** 10.1038/s41598-018-25774-1

**Published:** 2018-05-15

**Authors:** Annabelle Manalo, Alison K. Schroer, Aidan M. Fenix, Zoe Shancer, John Coogan, Tanner Brolsma, Dylan T. Burnette, W. David Merryman, David M. Bader

**Affiliations:** 1 Department of Cell and Developmental Biology, Vanderbilt University, Nashville, TN 37232 USA; 2 Biomedical Engineering, Vanderbilt University, Nashville, TN 37232 USA; 3 Division of Cardiovascular Medicine, Department of Medicine, Vanderbilt University, Nashville, TN 37232 USA

## Abstract

Centromere-binding protein F (CENP-F) is a very large and complex protein with many and varied binding partners including components of the microtubule network. Numerous CENP-F functions impacting diverse cellular behaviors have been identified. Importantly, emerging data have shown that CENP-F loss- or gain-of-function has critical effects on human development and disease. Still, it must be noted that data at the single cardiac myocyte level examining the impact of CENP-F loss-of-function on fundamental cellular behavior is missing. To address this gap in our knowledge, we analyzed basic cell structure and function in cardiac myocytes devoid of CENP-F. We found many diverse structural abnormalities including disruption of the microtubule network impacting critical characteristics of the cardiac myocyte. This is the first report linking microtubule network malfunction to cardiomyopathy. Importantly, we also present data demonstrating a direct link between a CENP-F single nucleotide polymorphism (snp) and human cardiac disease. In a proximate sense, these data examining CENP-F function explain the cellular basis underlying heart disease in this genetic model and, in a larger sense, they will hopefully provide a platform upon which the field can explore diverse cellular outcomes in wide-ranging areas of research on this critical protein.

## Introduction

Dilated cardiomyopathy (DCM), the most common form of cardiomyopathy in humans, can arise from mutations in over 50 genes^1^. There is expansive divergence in these genetic components as well as in the architectural abnormalities seen in the hearts of patients suffering from DCM^2–4^. We were the first to report an implication for any microtubule binding protein in the development of DCM^5^. With cardiac specific deletion of Centromere Protein F (CENP-F), a protein known to interact with microtubules^6–8^, the resulting animals exhibited enlarged ventricles with thinner walls, fewer trabeculae, increased fibrosis, and arrhythmia (all hallmarks of DCM), as well as death in 20% of outcomes^5^. While extensive work, including our own, has been reported on this pervasive disease, significant gaps in our understanding of the underlying alterations in cellular mechanisms driving dysfunction remain.

CENP-F is a very large (380 KD), multi-faceted protein with a myriad of cellular functions^9,10^. CENP-F is critical in mitosis and disruption of its function can result in mitotic delay and misalignment of chromosomes^11–14^. Additionally, CENP-F has been shown to have important interphase actions including regulation of ciliary function, vesicular transport, cell migration, cell shape, and organelle positioning^9,10,15^. A recurring theme in CENP-F function is interaction with the microtubule (MT) network and it is important to note that CENP-F loss of function has been shown to disrupt this cytoskeleton structure^9^. Recently and importantly, Waters *et al*.^15^ have identified a human mutation which leads to severe ciliopathy and microcephaly-related phenotypes leading to prenatal death. Conversely, over-expression of CENP-F also has lethal effects on the human condition as recent studies have demonstrated that CENP-F is the leading prognostic indicator of poor outcomes in specific forms of esophageal and breast cancers^16–18^. Even from this brief review of the literature, it is abundantly clear that gain and loss of CENP-F function can have devastating outcomes in development and disease.

From the work of several laboratories, it is clear that CENP-F is a critical and complex regulator of basic cellular function. Still, it should be noted that almost all reports, including our own, examining the effect of CENP-F loss of function at the unicellular level have used cells grown for a prolonged period in an *in vitro* setting. With this in mind, we used our genetic *CENP*-*F*^−/−^ murine model to isolate and immediately analyze fundamental properties of individual cardiac myocytes to determine whether and which myogenic properties might be changed with loss of CENP-F function. The *CENP*-*F*^−/−^ hearts from which these cells were derived demonstrated significant enlargement and ventricular dilation. Our data at the unicellular level demonstrate that loss of CENP-F function in cardiac myocytes leads to fundamental changes in the regulation of the cytoskeleton, contractile apparatus, cell junctions, mitochondrial positioning and shape, and calcium influx. Importantly, our studies also demonstrate *CENP*-*F*^−/−^ cardiac myocytes have loss of cellular stiffness while *CENP*-*F*^−/−^ fibroblasts isolated from the same hearts have amplified stiffness. These data reveal that different cell types with the identical disruption of CENP-F may respond differently to this genetic challenge. In this context, we also report human genomic studies linked to de-identified patient files identifying a CENP-F single nucleotide polymorphism (SNP) that is highly associated with human heart disease. Thus, the current data reveal the basic cell properties disrupted in cardiac myocytes with loss of CENP-F function and are the first to link CENP-F to human heart disease. In a broader sense, this study on the reaction of individual cells to loss of CENP-F may serve as a platform in the analysis of the numerous, diverse, and devastating responses of cells to fluctuation in CENP-F expression and function.

## Results

### Deletion of CENP-F disrupts the microtubule network in adult cardiac myocytes

**Figure 1:**
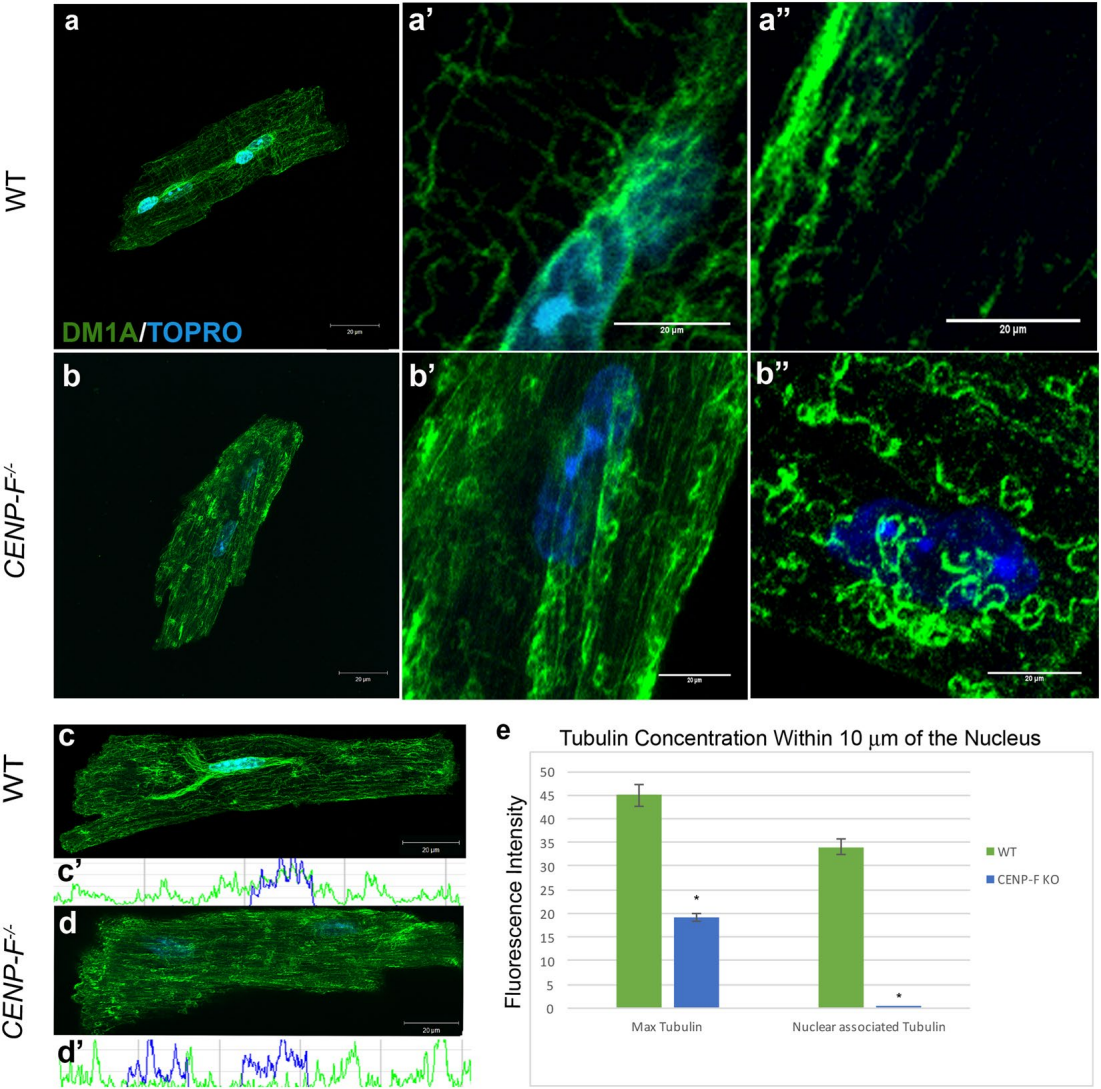
Microtubules (MTs) form rings at plus ends and lose connection with nucleus in *CENP*-*F*^−/−^ cardiomyocytes. Cardiomyocytes isolated from wild-type (**a**) and *CENP*-*F*^−/−^ (**b**) mice were fixed and immunostained with DM1A antibody (green) and TOPRO (blue). A zoom in of the circumferentially-oriented MT network (**a’**,**b’**) and longitudinal array of MTs (**a”**) are shown in wild-type and *CENP*-*F*^−/−^ mouse cardiomyocytes, respectively. Cardiomyocytes were treated with nocodazole to destabilize microtubules for phenotype comparison (**b”**). Immunostaining of MTs (green) and nucleus (blue) in wild-type cardiomyocytes show a microtubule-nuclei relationship (**c**), accompanied by a representative scan (**c’**). This relationship is completely gone in *CENP*-*F*^−/−^ cardiomyocytes (**d**), accompanied by a representative scan (**d’**). In (**e**), quantification of the maximum tubulin and nuclear associated tubulin of wild-type vs. *CENP*-*F*^−/−^ cardiomyocytes, show a tight relationship between microtubules and nuclei in wild-type cells opposed to a complete loss of association in *CENP*-*F*^−/−^ cells. In wild-type and *CENP*-*F*^−/−^ cells, n = 50 or more z-stack images analyzed for maximum tubulin. In wild-type and *CENP*-*F*^−/−^cells, n = 50 or more z-stack images analyzed for nuclear associated tubulin (**e**). Scale bars: 20 mm. *p < *0*.*001*. Error bars indicate SEM.

There are no reports on the effect of CENP-F loss of function at the level of the individual cardiac myocyte. Additionally, we found only one report analyzing the potential changes in the microtubule network in DCM^19^. Thus, in order to gain a clear understanding of the possible effect that loss of CENP-F may have on the cardiac microtubule network, a thorough characterization of microtubules in wild-type adult cardiac myocytes was first conducted using anti-alpha tubulin antibody DM1A. All cells reported below were analyzed within one hour after isolation from healthy or diseased hearts. Slices of z-stack images obtained through confocal microscopy were individually measured for microtubule density throughout the entirety of the cardiomyocyte. The microtubule network in these wild-type cardiac myocytes had a distinct and highly ordered pattern as seen in reconstructed z-stack images (Fig. 1). In agreement with previous studies on adult skeletal myocytes^20,21^, a concentration of the microtubule network around the nuclei of cardiac myocytes was observed (Fig. 1a,a’,c). Quantification of this association using Excel statistical analysis confirmed this result and demonstrated a high concentration of microtubules in association with the myocyte nucleus (Fig. 1e). In the non-nuclear domain, a lattice of individual longitudinally- and circumferentially-oriented microtubules was readily detected along the entirety of the cell (Fig. 1a’,a”). Again, this is in agreement with studies on skeletal myocytes that show microtubules distributed in this fashion along the entire length of the cell^22^. Thus, the organization of microtubules is largely preserved in both skeletal and cardiac myocytes. These results establish a baseline for comparing the potential effect that loss of CENP-F might have on the microtubule network in cardiac myocytes.

Supplementary Figure 1 shows the enlargement of the heart in the global knockout of CENP-F. This enlargement was observed in 100% of hearts examined from *CENP*-*F*^−/−^ animals and heart/whole body weights ratios also increased in the knockout group. Additionally, the ventricles were dilated and the ventricular wall was thinned (see Supplementary Fig. 1). In adult cardiac myocytes isolated from these *CENP*-*F*^−/−^ hearts, the overall structure of the microtubule network was severely disrupted. First, the concentrated association of the microtubules with the nucleus seen in wild-type myocytes was completely lost in knockout cells (Fig. 1b,d). The relationship of the microtubule network around the nuclei of wild-type and *CENP*-*F*^−/−^ cells was quantified using a point-line method on individual z-stack slices obtained by confocal microscopy (see Methods). These data confirmed the microtubule-nucleus relationship in wild-type cells (Fig. 1c,c’) and the nearly complete loss of this association in *CENP*-*F*^−/−^cardiomyocytes (Fig. 1d,d’; note the complete loss of overlap between blue (nuclear scan) and green (microtubule scan) in Fig. 1d’ which is seen in Fig. 1c’). Next, the non-nuclear component of the microtubule network was also disrupted with major aberrations. While the longitudinal array of non-nuclear microtubules was preserved, the circumferential component was reduced and significantly altered. The ends of these microtubules formed tight circles which were never seen in wild-type cardiac myocytes (Fig. 1b,b’). Interestingly, acute treatment of wild-type cardiac myocytes with nocodazole (a microtubule destabilizer) produced similar alterations of microtubules seen in adult *CENP*-*F*^−/−^ cardiac myocytes (Fig. 1b”). It should be noted that the curling/corkscrewing of microtubule ends in *CENP*-*F*^−/−^ myocytes produced a localized increase in immunofluorescent staining that appears randomly throughout the cell (see Fig. 1b,b’,b”). These data demonstrate that CENP-F loss of function has a significant and cell-specific (discussed below) effect on the microtubule network and are the first to show disruption of microtubules in any model of DCM.

### Basic cardiac myocyte structure is preserved in *CENP*-*F*^−/−^ cells but with an alteration at the cell junctions

**Figure 2:**
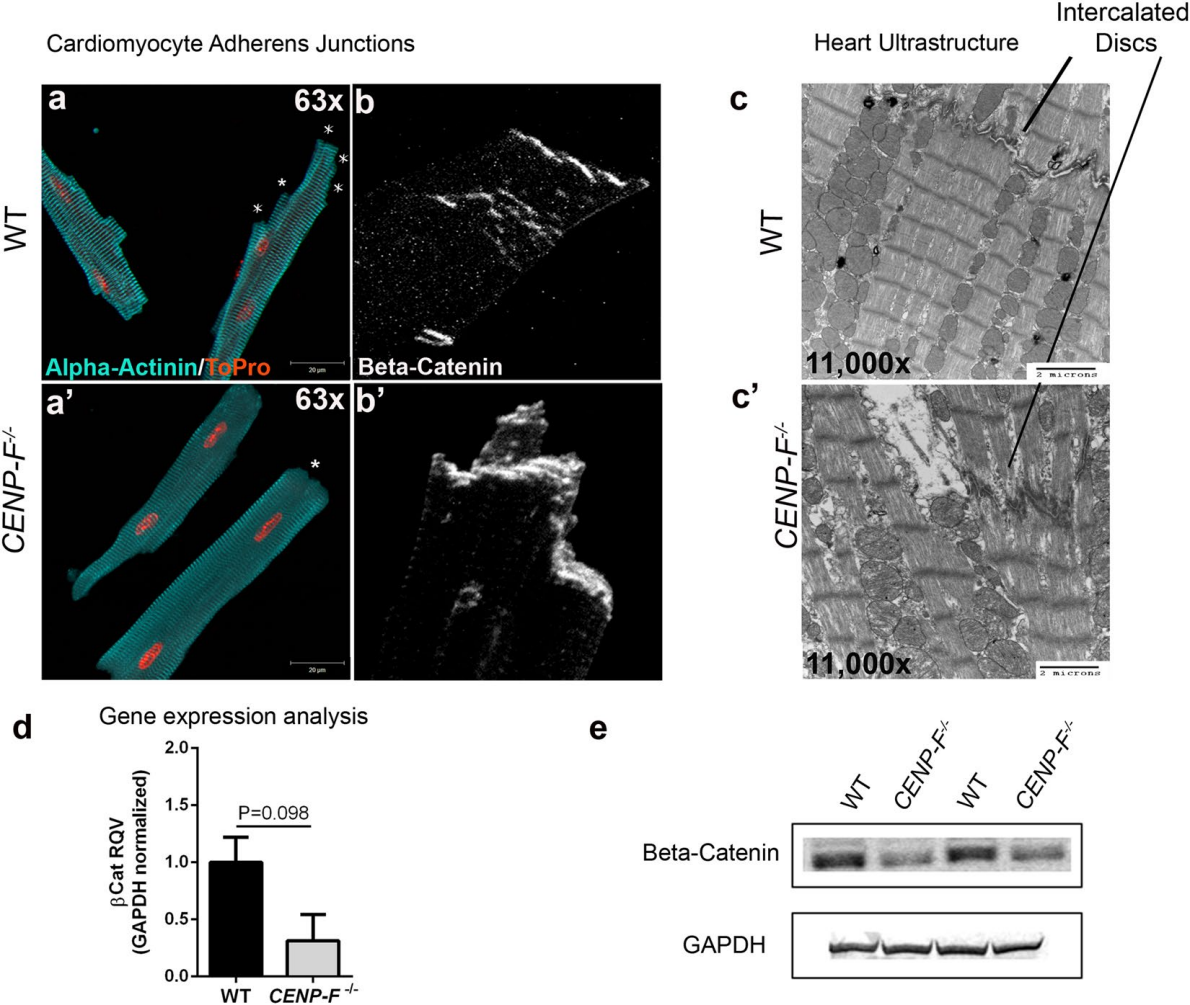
Intercalated disc organization is disrupted with the loss of CENP-F in cardiomyocytes. Cardiomyocytes isolated from wild-type (**a**) and *CENP*-*F*^−/−^ (**a’**) mice were fixed and immunostained with alpha-actinin 2 antibody (teal) and TOPRO (orange). In *CENP*-*F*^+/+^ cardiomyocytes, there are multiple junction ends, as indicated by * (**a**). However, in *CENP*-*F*^−/−^ cardiomyocytes, the cell termini is blunted, indicated by * (**a’**). The intercalated discs were immunostained with beta-catenin antibody (white) and the images are zoomed in to depict the lining of the cardiomyocyte junctions. In wild-type mice, there is thin and distinct junction staining (**b**) opposed to disintegrated and thickened junction staining of the mutant cardiomyocytes (**b’**). An EM image of wild-type heart shows a clear depiction of an intercalated disc, see horizontal structure pointed to by line (**c**), In *CENP*-*F*^−/−^ cardiomyocytes, the intercalated disc is highly disintegrated (**c’**). A gene expression analysis of beta-catenin in wild-type versus *CENP*-*F*^−/−^ cardiomyocytes (**d**). A western blot analysis of beta-catenin in wild-type versus *CENP*-*F*^−/−^ cardiomyocytes showed significant decrease in beta-catenin. 2 cropped samples are presented and n = 5, *p* < *0*.*05*. (Full-length gels are presented in Supplementary Fig. 4). Scale bars: (**a**) 20 mm; (**a’**) 20 mm; (**c**) 2 microns; (**c’**) 2 microns. Error bars in (**e**) represent standard error of the mean (SEM).

Intercalated discs connecting adjacent cardiac myocytes are essential for intercellular synchronization and transmission of force for proper contraction of the heart^23,24^. Kostetskii *et al*. have reported that disruption of the intercalated disc results in modest dilated cardiomyopathy^23^. In this context, the cell junctions in isolated wild-type and knockout cardiac myocytes were evaluated. First, an anti-alpha-actinin antibody (staining z-discs) was used to visualize the overall structure of these cardiac myocytes to provide context for the analysis of intercalated discs. In images of wild-type myocytes, the cell termini displayed a stacked/multilayered arrangement of myocyte/myocyte junctional domains in the terminal regions of the cells (Fig. 2a). Figure 2a demonstrates the generalized prominence of these cell termini that abruptly end perpendicular to the long axis of the myocyte (these domains indicated by * in this figure). Next, anti-beta-catenin staining was used to identify the adherens junction component of the z-disc and confirmed the multilayered arrangement in wild-type myocytes (Fig. 2b). Ultrastructurally, transmission electron microscopic (TEM) analysis further demonstrated the highly-ordered, well-defined structure of intercalated discs of wild-type cardiac myocytes (Fig. 2c).

While significant changes in z-disc structure were seen with loss of CENP-F function (discussed below), anti-alpha-actinin staining illustrated that the basic structure of the myocyte is maintained in the absence of CENP-F. However, the multi-layered structure of the cell termini of these cells was completely lost in the absence of CENP-F. The sharp, perpendicularly-arranged cell termini observed in wild-type cells were not seen in single cell analysis of *CENP*-*F*^−/−^ myocytes (Fig. 2a’). Instead, these myocytes have singularly-arranged cell termini (Fig. 2a’). Anti-beta-catenin staining was concentrated at this terminal domain and was not seen in the stepwise arrangement observed in wild-type cells (Fig. 2b’). Additionally, the staining in this single cell assay suggested that the terminal domain of *CENP*-*F*^−/−^ myocytes was thicker than their wild-type counterpart. TEM images of the intercalated discs of these myocytes showed that this terminal domain was widened and lacked the “sharpness” of discs observed in wild-type cells (Fig. 2c,c’). RT/PCR analyses were conducted on cell junction components, N-cadherin and beta-catenin, and decreased expression levels were noted for both gene products (Fig. 2d and Supplementary Fig. 2). Furthermore, western blot analysis of beta-catenin protein was performed and the cropped images revealed a significant decrease in this intercalated disc component of *CENP*-*F*^−/−^ myocytes as compared to wild-type cells (Fig. 2e and Supplementary Fig. 2). Full-length blots are presented in Supplementary Fig. 3. Therefore, although the beta-catenin images appear thickened, quantitative analyses at the RNA and protein levels show that these intercalated disc components are down-regulated with loss of CENP-F. Taken together, these data demonstrate the disruption of intercalated discs in hearts where DCM is induced with the loss of CENP-F function.

### Severe alteration of the sarcomeric network is observed in *CENP*-*F*^−/−^ hearts

To date, there are no reports linking the disruption of microtubule network components to structural deficits in the sarcomeric architecture in heart disease. Obviously, maintenance of the sarcomeric network is essential for cardiac function. To determine whether loss of CENP-F function impacted this fundamental unit of the cardiac myocyte, quantitative immunofluorescence was performed on isolated wild-type and knockout cardiac myocytes. Alpha-actinin antibodies were employed to mark the borders of sarcomeres (i.e., z-discs). Electronic microscopic analysis of myocytic architecture was also conducted in parallel.

**Figure 3:**
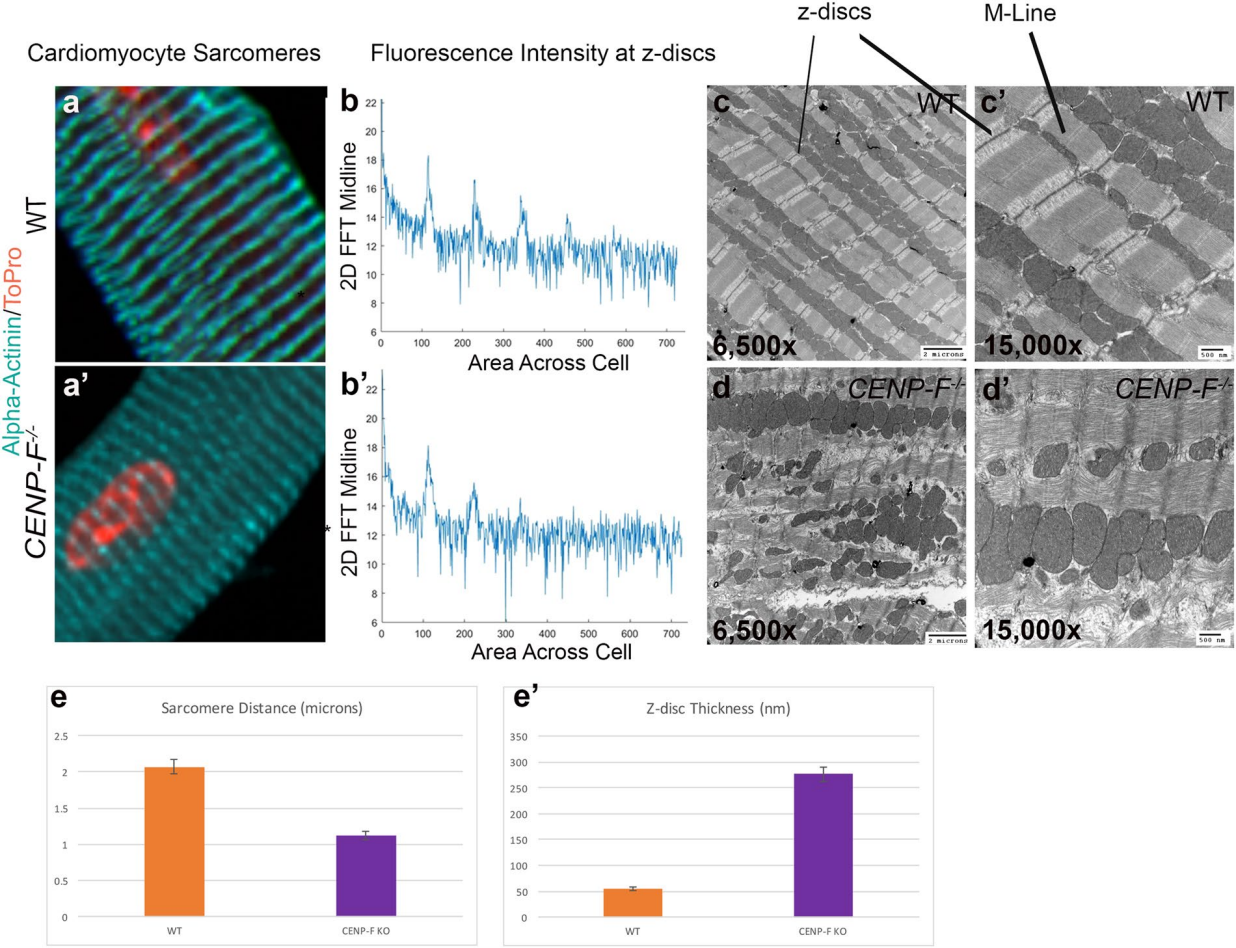
Sarcomere architecture is disrupted with the loss of CENP-F in cardiomyocytes. Cardiomyocytes isolated from wild-type (**a**) and *CENP*-*F*^−/−^ (**a’**) mice were fixed and immunostained with alpha-actinin antibody (teal) and TOPRO (orange). A zoom in view of the sarcomere structure displayed a distinct patterning in wild-type cardiomyocytes (a) versus a diffuse and thickened z-disc patterning in *CENP*-*F*^−/−^ cardiomyocytes (**a’**). A 2-D spectrum analysis of the fluorescence regularity in alpha-actinin stained cardiomyocytes displayed high intensity points at regular positioning in wild type cardiomyocytes (**b**). There is much more variability in the fluorescence staining of the *CENP*-*F*^−/−^ cardiomyocytes (**b’**). A TEM of a wild-type adult mouse heart shows the overall precise patterning of sarcomeric structure (**c**). The z-discs and M-lines are well defined, as depicted in a higher magnification (**c’**). In a CENP-F deleted mouse heart, the z-discs are disintegrated and there are breaks within the actomyosin network (**d**,**d’**). The distance between the z-discs is much shorter in *CENP*-*F*^−/−^ cardiomyocytes in comparison to the wild-type population, *p < *0*.*001* (**e**). Additionally, the width of the z-discs is much greater in the mutant cardiomyocytes opposed to the wild-type, *p < *0*.*001* (**f**).Scale Bars: (**c**) 2 microns; (**c’**) 500 nm; (**d**) 2 microns; (**d’**) 500 nm. Error bars in (**c**,**d**) represent standard error of the mean (SEM).

**Figure 4:**
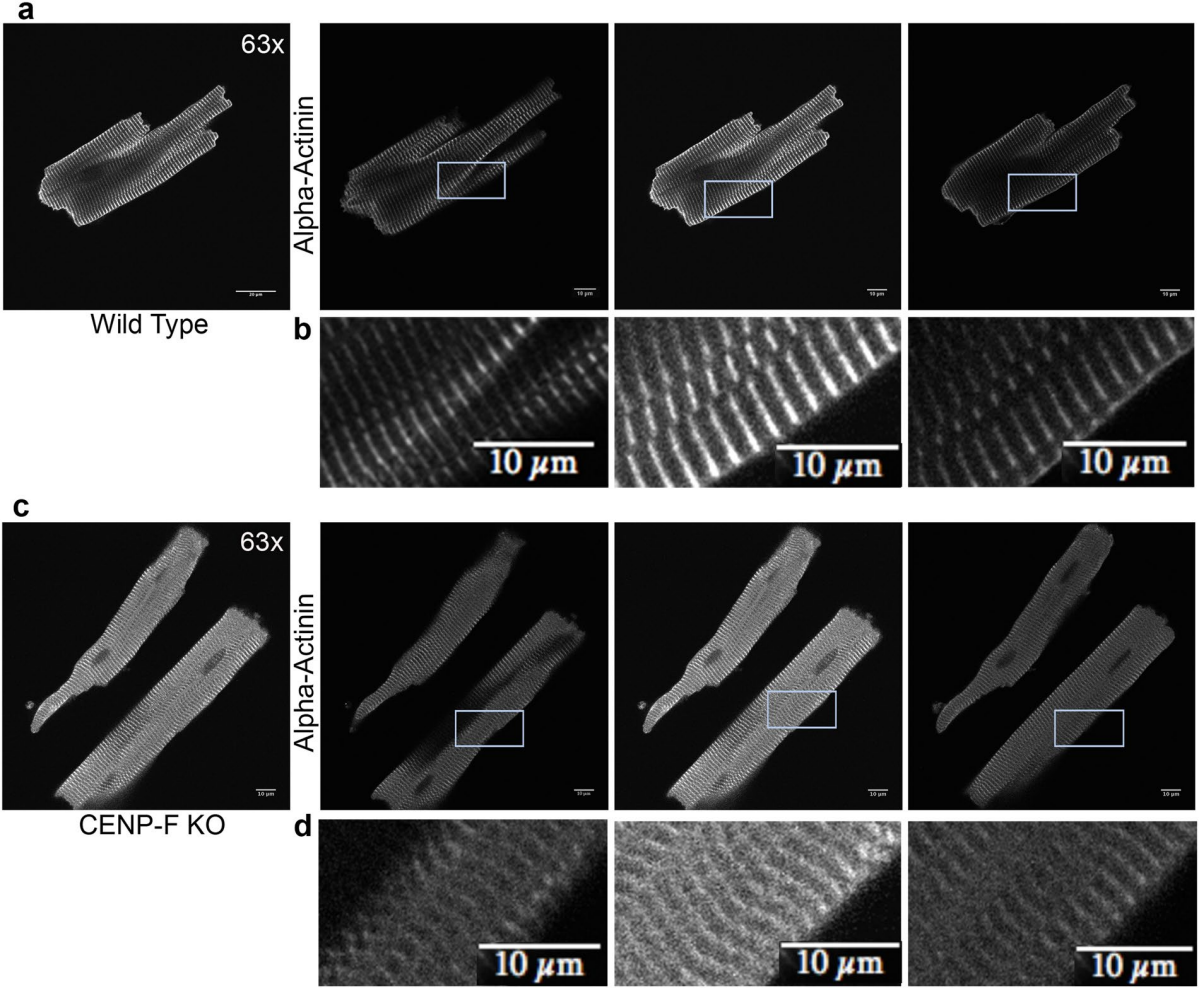
*CENP*-*F*^−/−^ cardiomyocytes have a disorganized z-disc structure compared to wild type cardiomyocytes. Individual slices of z-stack images in wild type cardiomyoctes show distinct z-disc staining with anti-alpha-actinin antibody (**a**,**b**). In *CENP*-*F*^−/−^ cardiomyocytes, alpha-actinin staining is not sharp/distinct. Additionally, z-discs are widened and severely disintegrated (**c**,**d**). This is in agreement with quantification of z-disc width conducted on TEM images (see Supplementary Fig. 4) All images are adjusted at the same brightness and contrast levels. Images are in greyscale for proper comparison of image quality. Scale bars: (**a**) 20 um; (**b**) 10 um; (**c**) 20 um; (**d**) 10 um.

Using confocal microscopy, the z-discs in wild-type myocytes displayed the distinctive periodic and recurring structure previously reported for this cell type with anti-alpha-actinin antibodies (Fig. 3a). These results were quantified using a 2-D fast fourier transform technique and showed precise periodic banding (Fig. 3b). Additionally, a breakdown of individual z-stack slices confirmed the precise spacing of z-discs seen in wild-type cardiomyocytes and the precision of this optical technique (Fig. 4a,b). An ultrastructural view of a wild-type heart also displayed crisp sarcomere structure with a distinct M-line, z-discs, and actomyosin network at lower (Fig. 3c) and higher (Fig. 3c’) power imaging.

Pervasive and widespread changes in the structure of the contractile apparatus were readily observed in *CENP*-*F*^−/−^ myocytes. Specifically, a very diffuse appearance of z-discs in the *CENP*-*F*^−/−^ cardiac myocytes was readily apparent when compared to wild-type cells at the light microscopic level (compare Fig. 3a,a’). 2-D fast fourier analysis of the alpha-actinin staining showed a lack of regularity in the z-discs of *CENP*-*F*^−/−^ cardiac myocytes (Fig. 3b’). A breakdown of individual z-stack slices confirmed that the blurred structure seen in *CENP*-*F*^−/−^ cardiac myocytes was of disintegrated z-discs rather than an issue of image quality (Fig. 4c,d). Additionally, a 3D movie further displayed the difference in z-disc precision and overall cardiomyocyte structure (Supplementary Movies 1 (WT) and 2 (KO)).

Electron microscopic imaging revealed dramatic changes in sarcomeric structure at the ultrastructural level with loss of CENP-F. TEM images of wild-type cells showed the precise and repeating structure of A and I bands with associated M lines and z-discs in highly ordered sarcomeres (Fig. 3c,c’). In contrast, sarcomeres of *CENP*-*F*^−/−^ myocytes lacked the sharp boundaries seen between A and I bands of the sarcomere seen in wild-type cells (Fig. 3d,d’ and Supplementary Fig. 4). Additionally, M lines were generally absent or highly distorted in these contractile structures and z-discs lacked the sharp, perpendicular arrangement seen in the normal structure. Accompanying these structural disruptions were two apparent changes in the sarcomere: shortening of the sarcomere and widening of the z-disc (Supplementary Fig. 4). Quantification of sarcomere length was conducted to determine whether quantitative decrease in sarcomere length did in deed occur with loss of CENP-F (see Methods for protocol; Supplementary Fig. 5 provides examples of images used in this quantification). As seen in Fig. 3e, significant shortening of sarcomeric length (*p < 0.005) was characteristic of knockout myocytes. These results are consistent with other reports on sarcomere shortening in other forms of cardiac disease^25,26^. The area and lineal persistence of z-discs in myofibrils of wild-type and knockout cells was also quantified using statistical analysis of ultrastructural images (Fig. 3e’ and Supplementary Fig. 4). These data confirm that z-discs of *CENP*-*F*^−/−^ myocytes were widened, lacked persistence, and were variable in shape with compared to the contractile apparatus in wild-type cells. Taken together, these data using different analytical tools demonstrate the essential nature of CENP-F in maintenance of the contractile apparatus.

### Mitochondria are misaligned and varied in size with the loss of CENP-F

**Figure 5:**
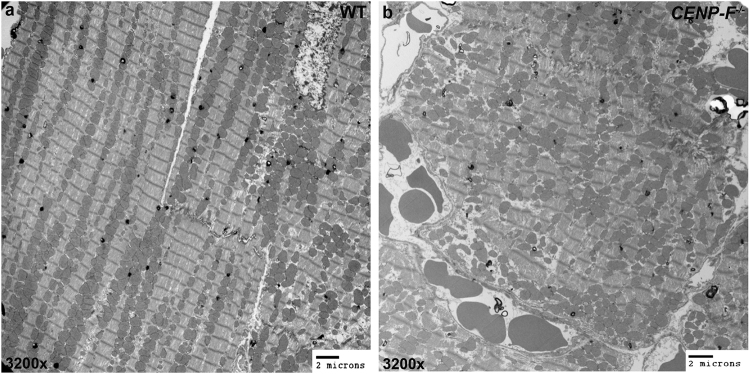
Mitochondria are misaligned and varied in size with the loss of CENP-F. Mitochondria are ordered, aligned, and relatively the same size in wild-type hearts (**a**). In *CENP*-*F*^−/−^ hearts, mitochondria have no alignment and are varied in size (**b**). Scale bars: (**a**) 2 microns; (**b**) 2 microns.

Recent reports have determined that mitochondrial disorganization is a precursor to DCM^27^. There is an intimate relationship between the structural integrity of the cytoskeleton and mitochondrial organization within a cardiac myocyte^28–30^. Having identified ultrastructural changes in *CENP*-*F*^−/−^ cardiac myocytes, transmission electron microscopic analysis was used to image and compare mitochondria in wild-type and knockout cardiac myocytes. As previously reported, the similarly sized mitochondria of wild-type mouse myocytes are aligned in long sequences between adjacent myofibrils^29^ (Fig. 5a). This arrangement was greatly disrupted in *CENP*-*F*^−/−^ hearts as mitochondria were rarely seen arranged in the long rows observed in wild-type myocytes (Fig. 5b). A quantification of mitochondrial persistence revealed an approximately 5-fold decrease in lineal persistence with the loss of CENP-F (Supplementary Fig. 6). Additionally, these misaligned mitochondria varied in shape and size in a random manner throughout the myocyte (Fig. 5b) (make sure the numbering is correct). These results are consistent with other models of DCM^27^ and, for the first time, link disruption of the microtubule network in the heart with abnormal mitochondrial size and alignment.

### CENP-F deletion results in softened cardiac myocytes and thus a reduction in mechanical force

There is increased fibrosis and loss of myocytes in DCM hearts that lead to overall myocardial stiffness^31^. Interestingly and conversely, recent reports have identified that isolated myofibrils and myocytes of hearts with specific forms of DCM have a decreased stiffness in comparison to wild-type cells^32,33^. While our previous report showed increased extracellular matrix in *CENP*-*F*^−/−^ hearts^5^, there are no studies that have directly examined the effect of CENP-F loss of function on the physical properties of single live cardiac myocytes.

**Figure 6:**
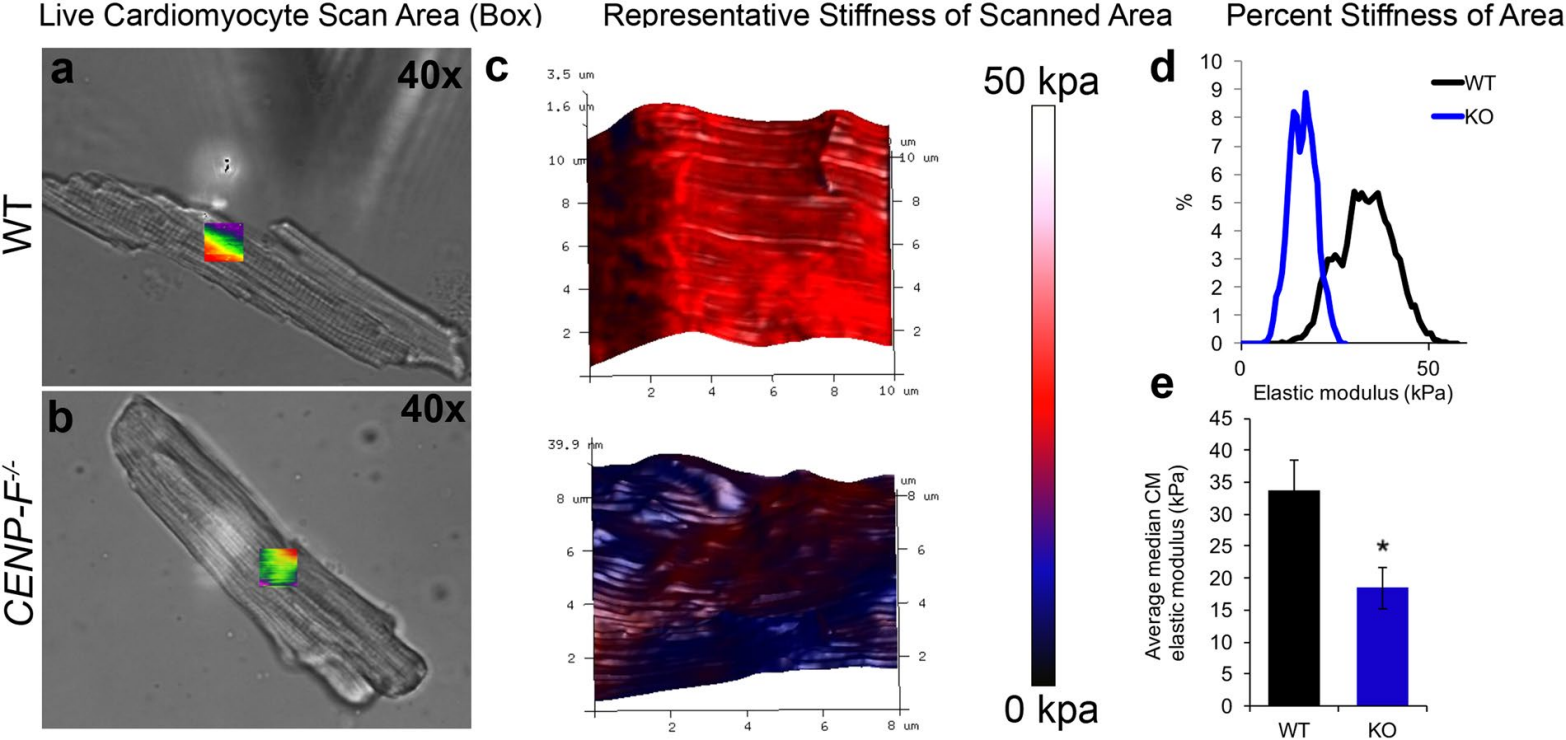
Cardiomyocytes are softened with the loss of CENP-F. Live cardiomyocytes isolated from wild-type (**a**) and *CENP*-*F*^−/−^ (**b**) mice were plated on laminin and analyzed for the elastic modulus of the cell surface. Representative topography plots (color bar) show a stiff surface across the 3D rendering of the wild-type cardiomyocyte (red), while, the *CENP*-*F*^−/−^ cardiomyocyte (blue) is significantly softened (**c**). Distribution of cell stiffness from representative scans (**d**). The average median calculation of the elastic modulus of wild-type vs. *CENP*-*F*^−/−^ cardiomyocytes is measured less (kpa) by an approximate 2-fold difference, *p* = *0*.*0124* (**e**). Error bars in (**e**) represent the standard error of the mean (SEM).

To measure cell stiffness, Atomic Force Microscopy (AFM) was performed on wild-type and *CENP*-*F*^−/−^ cardiac myocytes immediately after isolation. A 10 × 10 micron area of the cell was scanned for cell stiffness (Fig. 6a). In wild-type cardiac myocytes, there was a consistent stiffness of the scanned area (Fig. 6c,d). The cell recorded a stiffness of approximately 35 kpa (Fig. 6e.) The stiffness (elastic modulus) of the *CENP*-*F*^−/−^ cardiac myocytes was significantly less than that of wild-type cells (Fig. 6c–e). Additionally, stiffness fluctuated in different areas of the knockout cell which was not observed in wild-type cells (Fig. 6c). Further, these cells were unable to withstand the ongoing tapping of the cantilever for longer than 1 hour which wild-type cells were able to endure. Interestingly and conversely, concurrent AFM analysis of cardiac fibroblasts isolated from the same *CENP*-*F*^−/−^ hearts detected stiffness not observed in the wild-type population (Supplementary Fig. 7). Thus, cardiac myocytes and fibroblasts exhibit opposite reactions in terms of cell stiffness with the same genetic mutation.

### Calcium influx is decreased in *CENP*-*F*^−/−^ cardiomyocytes

**Figure 7:**
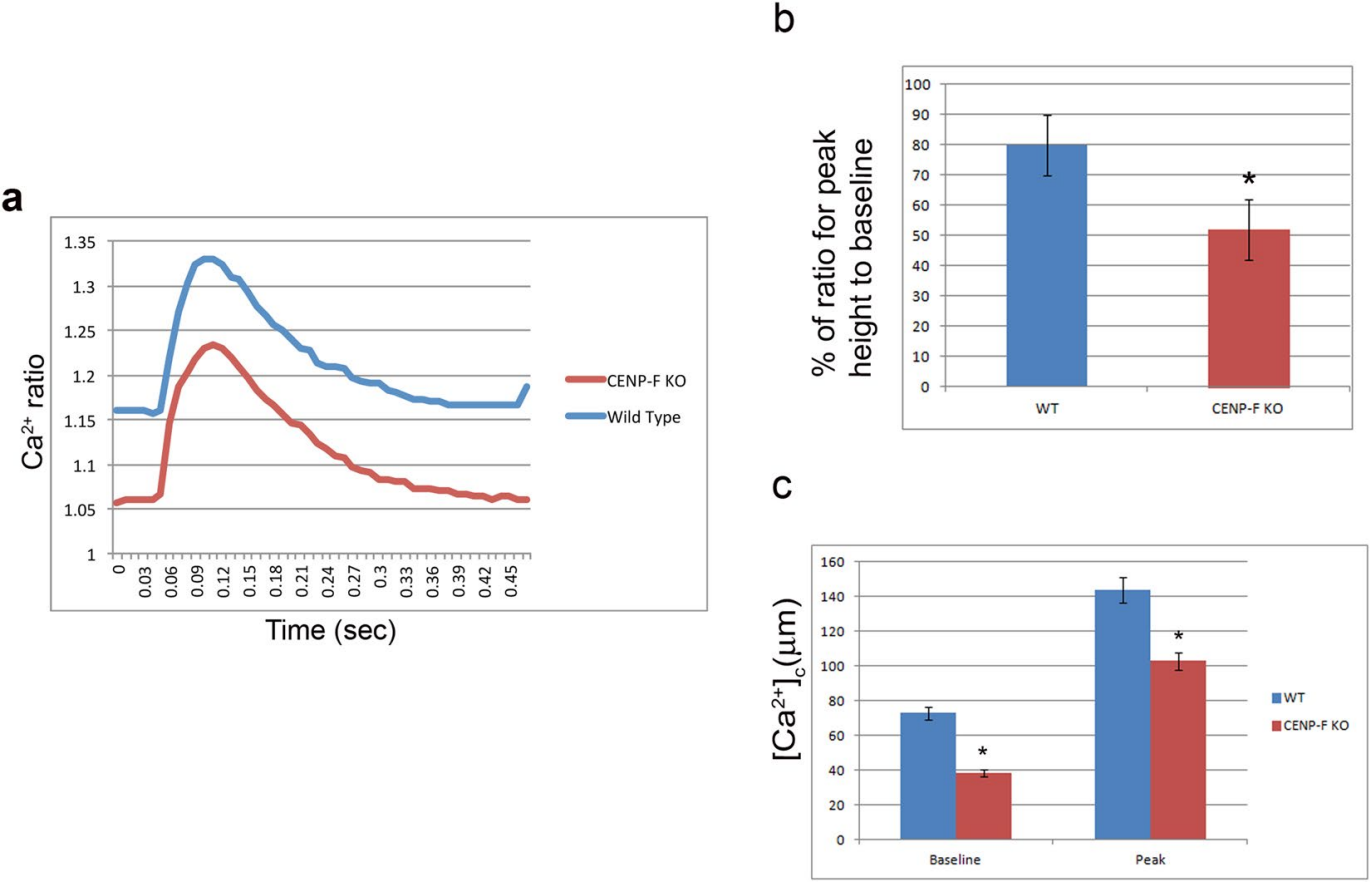
Calcium transients are decreased in *CENP*-*F*^−/−^ live cardiomyocytes. Physiological analysis of live cardiomyocytes was conducted using Ionoptix Experimentation. Fluorescence ratio of FURA-2 binding to calcium was recorded from myocytes isolated from wild-type and *CENP*-*F*^−/−^ hearts. Calcium ratio was measured during contraction/relaxation cycle resulting in a decreased ratio in *CENP*-*F*^−/−^ cardiomyocytes (**a**). Calcium released during the contraction/relaxation cycle was decreased in *CENP*-*F*^−/−^ cardiomyocytes, *p < *0*.*001* (**b**). In phases of the transient, both the pre-stimulation baseline value and maximal deflection from baseline (peak) was less, *p < *0*.*05* (**c**). Each bar represents mean SEM, n = 5–10 cardiomyocytes.

In heart failure, calcium entry and removal are critical functions that are severely altered affecting cardiac action potential^34^. In turn, the ability of heart to adequately pump sufficient blood is lost. The gravity of cellular parameters that are disrupted with loss of CENP-F led us to conduct a functional evaluation of isolated individual wild-type and *CENP*-*F*^−/−^ cardiac myocytes. During the contraction-relaxation cycle, the calcium transients of single wild-type and knockout cells were measured using Fura-2AM calcium detection dye with Ionoptix. These studies revealed that the calcium transients in *CENP*-*F*^−/−^ myocytes were significantly decreased when compared to wild-type cells (Fig. 7a). In conjunction to these results, significant changes in parameters that influence the calcium influx were seen. The percent of calcium released over multiple phases was decreased in *CENP*-*F* myocytes (Fig. 7b). The overall baseline peak was greatly lessened in each transient phase (Fig. 7c).

#### Footnote

Concurrent Ionoptix analysis of contraction suggested differences in wild-type and knockout cells but variability in the analysis of individual cells precluded any definitive conclusion that significant loss of contractile function occurred with loss in regulation of calcium influx.

### Loss of CENP-F is associated with human heart failure

CENP-F is the first microtubule binding protein to be linked to heart disease in a model organism. Given the diversity of genetic backgrounds influencing cardiac disease in humans, a PheWAS Manhattan Plot Study was performed to correlate the results of the *CENP*-*F*^−/−^ mice hearts with that of humans. In a biorepository that links 25, 579 patients at Vanderbilt University Medical Center, CENP-F single nucleotide polymorphisms (snps) were probed for their association with heart failure in patient populations (Supplementary Fig. 8). One common variant (MAF 45%) in CENP-F, rs7289, was associated with Heart Failure with reduced Ejection Fraction (HFrEF) (OR = 1.12, 95% CI 1.04 to 1.20, q = 0.09). This variant encodes an amino acid substitution (N3106K), which is in the farnesylation domain/motif of CENP-F. This domain is located directly adjacent to the microtubule binding domain of CENP-F at the C-terminus^35^, and farnesylation of CENP-F is key for the localization of CENP-F to the nuclear envelope and for CENP-F degradation or turnover^35^ (Supplementary Fig. 8). This targeted PheWAS study suggests that common variants in CENP-F may modulate risk of heart failure with systolic dysfunction in humans and is consistent with the systolic dysfunction identified in *CENP*-*F*^−/−^ murine hearts^5^.

## Discussion

The current study addresses two interrelated and evolving areas of study: the genetic basis of cardiovascular disease and the regulation of basic cell function by CENP-F. These data reveal the fundamental reaction of cardiac myocytes to loss of CENP-F function and lay the groundwork for future studies focusing on individual cellular components in diverse cell types regulated by this complex and multifunctional protein.

Dilated cardiomyopathy (DCM) is a devastating disease with diversity and complexity in its genetic origins and subsequent cellular responses causing approximately 10,000 deaths and 46,000 hospitalizations each year in the United States alone. Interestingly, while more than 50 genes have been linked to this disease^1^, no gene encoding a microtubule-binding protein had been identified as a cause of DCM until our recent study^5^. All the changes we observe at the organ, cell, and ultrastructural levels in our current study suggest that this genetic model of CENP-F loss of function reflects previous genetic studies leading to a form of DCM. Thus, we discuss our findings in the context of DCM as the classic signs of the disease are detected in this mouse model. Importantly, the underlying cellular and sub-cellular structural changes resulting from loss of CENP-F function which result in this form of DCM had not been determined until the current study. Thus, in-depth analyses of single, isolated individual were needed for a comprehensive understanding of this model of DCM and, in a larger sense, to understand the potential role of microtubule network dysfunction in cardiac disease.

The current data generated from analysis of individual wild-type and knockout cardiac myocytes clearly demonstrate that loss of CENP-F function results in significant disruption of the microtubule network. Analysis of microtubule network structure in all genetic models of heart disease has been very limited to this point^19^. With loss of CENP-F function, the perinuclear domain of the microtubule network in myocytes was completely lost. In the non-nuclear domain of the network, the longitudinal domain remained intact but its horizontal/lateral component was disrupted and replaced by highly coiled ends. Importantly, a disorganization of the grid-like microtubule network of skeletal myocytes is also seen in Duchenne muscular dystrophy^36^. Conversely, it is interesting and important to note that the one study focused on MT network structure in DCM reported that no differences in the density or distribution of MTs were detected in their tachycardia-induced DCM^19^. While our current studies do not reveal a molecular mechanism leading to this heart disease, disruption of CENP-F is known to negatively impact a myriad of molecular interactions governing microtubule regulation of basic cell functions^5–14^. Thus, retrospectively and going forward, it will be interesting to determine whether disruption of the microtubule network is a hallmark of DCM, is only associated with disruption/mutation of microtubule components, or is restricted to disruption/mutation of CENP-F alone. Further, given that DCM is a frequent occurrence in chemotherapeutic treatment of cancers and that many of these drugs target microtubules^37,38^ careful, broad, and ongoing investigation of the role of microtubule network dysfunction in cardiac disease may be warranted.

Confocal, immunochemical, and electron microscopic analyses of individual *CENP*-*F*^−/−^ myocytes clearly demonstrate dramatic alterations in overall cell structure, especially in contractile, junctional, and mitochondrial domains when compared to normal myocytes. These changes were quantitatively significant and underlie physiological defects noted in this genetic model of disease. Disruption of the myofibril and its sarcomere components is a common phenotype in DCM and can be caused by mutation of genes coding for both sarcomeric and non-sarcomeric proteins^2^. Additionally, breakdown of the intercalated discs with concurrent decrease in N-cadherin and beta catenin expression is seen in *CENP*-*F*^−/−^ myocytes (Fig. 2 and Supplementary Fig. 2) and is consistent with the report demonstrating that loss of N-cadherin leads to disc aberration^23^. The mitochondrial disruption seen with loss of CENP-F function is also a trait of several forms of DCM resulting from mutations in the mitochondrial or nuclear genomes^39^. While we found only one other publication attempting to decipher the functional role of microtubules in the heart, physiological disruption of calcium influx in single cells with loss of CENP-F also demonstrates the functional relevance for a microtubule associated protein in the heart physiology (Fig. 7). Taken together, it is important to reiterate that in this first model of cardiac disease resulting from loss of a microtubule-binding protein, these hallmark disturbances^2,24,37,38^ and the resulting “final common pathway” that is DCM are observed. But, at the same time, it is important to emphasize that loss of CENP-F function is not identical to any specific form of heart disease previously reported and that loss of other microtubule-associated proteins may or may not produce the phenotypes seen here.

It is important to note that differing models of CENP-F disruption can lead to differing results with the same cell type. For example, the field has reported varying *in vitro* results concerning the promotion of aneuploidy with disruption of CENP-F; one model using RNA interference technologies demonstrated resulting aneuploidy^13^ while another genetic deletion model did not^9^. The present study is the first to examine the reaction of two different cell types, isolated from the same organ, with identical and simultaneous disruption of gene function. Deletion of CENP-F in this genetic model results in cardiac myocytes displaying a more softened profile in atomic force analyses, whereas cardiac fibroblasts isolated from the same hearts exhibited a stiffened phenotype (Fig. 6). Revealing this diversity of reaction may be specifically important to the cardiovascular field, as heart wall stiffening is widely observed in DCM^4,31^. Knowing which cells and tissues underlie this phenotype may prove of importance in future therapeutic strategies. In a broader sense, the current data demonstrating the exact opposite responses of two cell types derived from the same organ to loss of CENP-F function would predict a diversity of response of other cell types in reference to the many and varied cellular behaviors governed by this gene product.

## Materials and Methods

### Mouse lines

Mice were previously described^8^. *CENP*-*F*^*fl*/-^, *CMV*-*CRE* animals (The Jackson Laboratory, Bar Harbor, ME) were crossed to generate *CENP*-*F*^−/−^; *CMV*-*CRE animals*. *CENP*-*F*^+/+^; *CMV*-*CRE* animals were used to generate control cardiac myocytes. Cardiac myocytes were isolated from adult mice aged 3–4 months to assure maturity. All animal care and experimental procedures were approved and performed in accordance with the ethical standards set by the Institutional Animal Care and Use Committees of Vanderbilt University and by the National Institute of Health (NIH).

### Cardiac Myocyte Isolation

Adult *CENP*-*F*^+/+^; *CMV*-*CRE* and *CENP*-*F*^−/−^; *CMV*-*CRE* mice were terminally anesthetized by inhalation of isofluorane five minutes after heparin injection. Hearts were suspended from a Langendorff perfused column via cannulation of the aorta and perfused with cell isolation buffer containing in mM: NaCl 113, KCl 4.7, MgSO_4_ 1.2, Na_2_HPO_4_ 0.6, KH_2_PO_4_ 0.6, NaHCO_3_ 12, KHCO_3_ 10, Taurine 30, HEPES 10 to which Liberase TM 2.7 mg (Roche cat.#05 401 127 001). Tissue was then minced and subject to further digestion followed by CaCl_2_ dilution and bovine serum albumin (2 mg ml^−1^ until myocytes were liberated easily by titration with a sterile Pasteur pipette and filtered). Cardiac myocytes were then reintroduced to physiological CaCl_2_ in increasing increments. Experiments were performed at 37 degrees C. Cardiac myocytes were plated on Laminin (10micrograms/ml) coated dishes and incubated for a minimum of one hour prior to manipulation or fixation with 4% paraformaldehyde. After a 15 minute fixing, cardiac myocytes were washed with 1x PBS and stored at −20 degrees C for further observation.

### Cardiac Fibroblast Isolation

Adult *CENP*-*F*^+/+^; *CMV*-*CRE* and *CENP*-*F*^−/−^; *CMV*-*CRE* mice were terminally anesthetized by inhalation of isofluorane five minutes after heparin injection. Hearts were suspended from a Langendorff perfused column via cannulation of the aorta and perfused with cell isolation buffer containing in mM: NaCl 113, KCl 4.7, MgSO_4_ 1.2, Na_2_HPO_4_ 0.6, KH_2_PO_4_ 0.6, NaHCO_3_ 12, KHCO_3_ 10, Taurine 30, HEPES 10 to which Liberase TM 2.7 mg (Roche cat.#05 401 127 001). Tissue was then minced and subject to further digestion followed by CaCl_2_ dilution and bovine serum albumin (2 mg ml^−1^ until myocytes were liberated easily by titration with a sterile Pasteur pipette and filtered). The supernatant containing nonfibroblasts were removed and plated on 4 well slides or uncoated plates for further observation.

### Antibodies for Immunofluorescence

Cardiac myocytes were fixed for 15 minutes at 37 degrees Celsius in 4% paraformaldehyde with 0.01% Triton-X100 prior to antibody labeling:

DM1A: mouse monoclonal (ab7291; Abcam).

Alexa Fluor 488 Phalloidin (A 12379; Invitrogen, Carlsbad, CA).

Alpha-Actinin (A7811; Sigma-Aldrich).

Beta-Catenin (C7207; Sigma-Aldrich).

TO-PRO-3 Iodide (T3605; ThermoFisher Scientific) was used to visualize DNA.

MF20: mouse monoclonal (Bader Laboratory, also available through Developmental Studies Hybridoma Bank).

### Confocal Microscopy

Cells were imaged on Zeiss LSM 510 Meta inverted confocal microscope in the Vanderbilt Cell Imaging Shared Resource Core at 40× (Plan-NEOFLUAR OIL), 63× (Plan-APOCHROMAT OIL), and 100× (Plan-NEOFLUAR OIL). Projections were created from 0.3 micrometer slices through the full thickness of the cardiomyocytes. The imaging mode used was a 3-D “Z-series” technique.

### Quantification of Microtubule/Nuclear Relationship

From z-stack confocal images of cardiomyocytes, individual slices were analyzed using a point-line method in ImageJ^40^. The line was specifically drawn throughout the nucleus from each end of the cardiomyocyte. The fluorescence of DM1A (microtubules) and TOPRO (nuclei) was calculated in ImageJ. The relationship of microtubule- and nuclear-associated fluorescence was quantified from analysis of 50 or greater individual fluorescence images per group. The tubulin concentration within 10 micrometers of the nucleus relative to the maximum tubulin concentration found throughout the cell was measured for specificity of the relationship and to avoid any imaging noise. Statistical analysis was determined using the t-test tool in Excel programming, in which, identification of the significance of the manually entered data points was retrieved. From the t-test, calculation of the P-value was generated.

### Western Blot Analysis

For Western blot, pelleted *CENP*-*F*^+/+^ and *CENP*-*F*^−/−^ cardiomyocytes were solubilized in Laemmli sample buffer in the presence of protease inhibitors^41^. Equivalent amounts of extracts were run on 3–8% SDS gels using standard methodologies and transferred to nitrocellulose paper in the presence of 20% methanol, 10 mM β-mercaptoethanol, and 0.1% SDS. Blots were washed with phosphate-buffered saline (PBS) and blocked with 5% bovine serum albumin (BSA) in PBS for 1 h, followed by a 1-h incubation with anti–beta-catenin antibody and diluted 1:3000 in 5% BSA/PBS. Blots were washed in PBS with 0.1% Triton X-100, followed by reaction with goat anti-mouse (IRDye 800CW; Li-Cor) and similar washing for imaging on an Odyssey system. Controls for loading equivalence were conducted under similar conditions with GAPDH. For the western blot statistical analysis, a densitometry scan was performed in Imagej on images acquired in Odyssey. A t-test was then generated on Excel programming.

### PheWAS Study

To determine whether CENP-F is relevant to cardiac dysfunction in humans, we utilized BioVU, the Vanderbilt University Medical Center biorepository that links a de-identified version of the electronic health record to DNA samples and genotype data^42^. The study cohort included 25,579 adults of European descent genotyped on the Illumina HumanExome BeadChip array v.1.0 (exome chip) as part of several BioVU research initiatives (Supplementary Table 1). The exome chip contains ~250,000 rare and common coding single nucleotide polymorphisms (SNPs), including 26 missense variants in CENP-F with a minor allele frequency >0.05% (http://genome.sph.umich.edu/wiki/Exome_Chip_Design). Variants in CENP-F were tested for association with heart failure with reduced ejection fraction (HFrEF) in BioVU using a phenome-wide association study (PheWAS) based phenotype (PheCode 428.3). PheWAS is a validated approach to interrogate the medical phenome that uses a validated, curated medical phenome that hierarchically groups International Classification of Disease (ICD-9) billing codes into ~1,800 phenotypes, each with defined control groups^43^. Variants in CENP-F were tested for association with HFrEF using logistic regression with an additive model adjusted for age and sex, and analyses were restricted to individuals of white European descent to limit population stratification. The Benjamini-Hochberg correction was applied to account for multiple testing with a q < 0.1 considered statistically significant.

Clinical cohorts genotyped on exome chip

1. An elderly cohort (patients over age 75) with at least 3 years of follow-up
2. A rare-diseases cohort, including patients with FDA-defined rare diseases (no cardiomyopathies are included among the FDA-defined rare diseases)
3. A healthy longitudinal cohort including patients with 1–2 notes per year for at least 5 years
4. A sick longitudinal cohort including patients with 3–6 notes per year for at least 5 years
5. The Vanderbilt Electronic Systems for Pharmacogenomic Assessment Cohort, created to investigate drug response phenotypes^3^
6. A cancer cohort consisting of patients within the Vanderbilt Tumor Registry
7. A pediatric cohort consisting of patients under 18 years of age with at least one pediatric visit

### Quantification of Cardiac Myocyte Mitochondrial Persistence

For further analysis of mitochondrial population in EM images, a box was placed over a given area and assessed for mitochondrial persistence in Imagej^40^. In five or more images, a line was drawn through the number of mitochondria in a row. The differences in mitochondrial persistence were quantified using the t-test tool in Excel programming.

### Quantification of Cardiac Myocyte Sarcomere Structure

Using Imagej, a fast Fourier transform was run on a trace along the length of the sarcomere structure from a series of z-stack images of cardiac myocytes stained for alpha-actinin. The position of the peak of the normalized phase plot was used to calculate the sarcomere length. Statistical analysis was performed using the t-test tool in Excel programming. A 2D FFT transform was run to observe differences in the frequency distribution, plotted as a 2D power spectrum. Sarcomere length in wild-type and knockout myocytes was also determined at the TEM level. Distances were measured from the midline of adjacent z-discs in myofibrils where at least four sarcomeres were displayed in the section. Over 100 sarcomeres were measured in non-adjacent sections from three hearts in each group. The measurements were analyzed with the t-test tool in Excel programming. For further analysis of z-discs in EM images, a 1.5′ × 0.12″ box was placed over 50 or more individual z-discs in Imagej^40^. If any part of the z-disc could not fit within the area of the box, the z-disc was considered disintegrated. Additionally, in twenty images or more, a line was drawn through the number of z-discs in a row. The differences in z-disc structure and linear persistence were quantified by using the t-test tool in Excel programming.

### Transmission Electron Microscopy

Hearts were removed from adult mice and immediately perfused in 2.5% gluteraldehyde in 0.1 M cacodylate buffer, pH7.4 at room temperature (RT), then further dissected for optimal transmission electron microscopy processing. The samples were washed in 0.1 M cacodylate buffer, then incubated 1 hour in 1% osmium tetraoxide at RT, then washed with 0.1 M cacodylate buffer, followed by a 30-minute incubation in 1% potassium ferrocyanide for additional membrane contrast. Subsequently, the samples were dehydrated through a graded ethanol series and then 3 exchanges of 100% ethanol. Next, the samples were incubated for 5-minutes in 100% ethanol and propylene oxide (PO) followed by 2 exchanges of pure PO. Samples were then infiltrated with 25% Epon 812 resin and 75% PO for 30 minutes at RT. Next, they were infiltrated with Epon 812 resin and PO [1:1] for 1 hour at RT then overnight at RT. Next day, the samples went through a [3:1] (resin: PO) exchange for 3–4 hours, then incubated with pure epoxy resin overnight. Samples were then incubated in two more changes of pure epoxy resin than allowed to polymerize at 60 °C for 48 hours. 70–80 nm ultra-thin sections were cut and collected on 300-mesh copper grids and post-stained with 2% uranyl acetate and then with Reynold’s lead citrate. Samples were subsequently imaged on the Philips/FEI Tecnai T12 electron microscope at various magnifications.

### Quantification of Cardiac Myocyte Stiffness by AFM

A Bruker biocatalyst AFM system was used to measure the stiffness of isolated cardiac myocytes, cardiac fibroblasts and MEFs. The quantitative nano-mechanical mapping mode was used to assess both the topography and elastic modulus of wild-type and *CENP*-*F*^−/−^ cells. This mode uses a blunted pyramidal tip to indent and gather mechanical information from about a micron below the cell surface. Between 4 and 10 cells were scanned per group and the median elastic modulus calculated from 1–2 approximately 10 × 10 micron scans for each cell (>10,000 measurements per scan). The average median cell stiffness was calculated and compared between wild-type and *CENP*-*F*^−/−^ with student t-test in Excel programming.

### RNA isolation

RNA was isolated using mRNeasy mini kit (Qiagen) following manufacturer instructions and was quantified using Take3 micro-volume plates with Synergy Mx plate reader and Gen5 software.

### Real-Time PCR

Total RNA was reverse transcribed, using TaqMan RT kit (Applied Biosystems) with random hexamers following manufacturer instructions. Individual transcript levels were quantified with real-time PCR TaqMan probes, and relative quantitation values (RQV) were calculated with normalization to the housekeeping gene, GAPDH. Statistical analysis was performed using t-test in Excel programming.

### Measurement of Intracellular Ca

Cardiomyocytes were loaded with Fura-s acetoxymethyl ester, Fura-2AM (Molecular Probes Inc, Eugene, OR) as described previously. After an eight minute incubation in Fura-2AM at room temperature, cardiomyocytes were washed twice for 10 minutes with Tyrode’s solution. Fura-2AM loaded Ca transients were recorded during spontaneous beating or 0.2 Hz field stimulation in 2 mmol/L Ca Tyrode’s solution. Ca transients were recorded and analyzed using commercially available data analysis software (IonOptix, IonWizard Milton, MA). All experiments were conducted at room temperature. Tyrode’s solution containing (in mmol/L): CaCl_2_, NaCl 134, KCl 5.4, MgCl_2_ 1, Glucose 10, and HEPES 10, pH adjusted to 7.4 with NaOH.

### Data availability statement

All data generated or analyzed during this study are included in this published article (and its Supplementary Information files).

## Electronic supplementary material

Supplementary Figures 1–8

Supplementary Movie 1

Supplementary Movie 2
