# Supplementary material for: Loss of CENP-F Results in Dilated Cardiomyopathy with Severe Disruption of Cardiac Myocyte Architecture
Source: Sci Rep. 2018 May 15;8:7546. doi: 10.1038/s41598-018-25774-1 (PMC5953941; doi:10.1038/s41598-018-25774-1)
Supplement: Supplementary file 1 — Supplementary Figures 1–8 [file 41598_2018_25774_MOESM1_ESM.pdf]

Loss of CENP-F Results in Dilated Cardiomyopathy with Severe Disruption of Cardiac Myocyte Architecture

Annabelle Manalo[1], Alison K. Schroer[2], Aidan M. Fenix[1], Zoe Shancer[3], John Coogan[3], Tanner Brolsma[3], Dylan T. Burnette[1], W. David Merryman[2], David M. Bader[1,3*]

Supplementary Figures

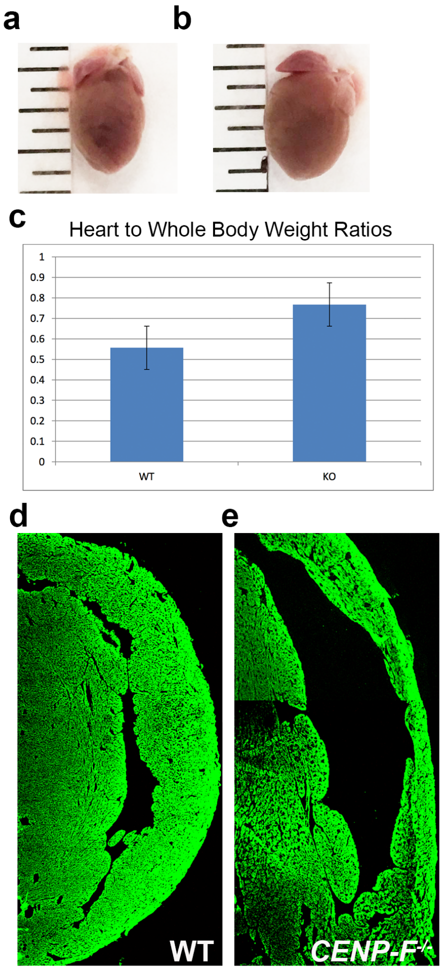

Supplemental Figure 1. The loss of CENP-F leads to enlargement/dilation of hearts in adult mice.
Isolated adult hearts from wild-type **(a)** and *CENP-F*$^{-/-}$ **(b)** mice are measured in inches. All hearts (100%) isolated from *CENP-F*$^{-/-}$ animals were enlarged when compared to hearts taken from wild-type mice **(b)**. *n=10* per group. Ten animals per group were weighted as were their hearts after euthanasia and immediate extraction. Percent heart to whole body weight is given for wild-type and *CENP-F*$^{-/-}$ groups and this ratio was increased in the *CENP-F*$^{-/-}$ group **(c)** *n=10.* *$p<0.005$. Error bars represent SEM; statistical analysis was performed using the TTEST tool in Excel programming**.** A composite image of MF20 stained tissue sections displaying the right ventricle of an adult wild-type heart **(d)**. A composite image of MF20 stained tissue sections of the right ventricle in an enlarged *CENP-F*$^{-/-}$ heart, demonstrates ventricular dilation and a thinned wall **(e)** *n=10.*

a

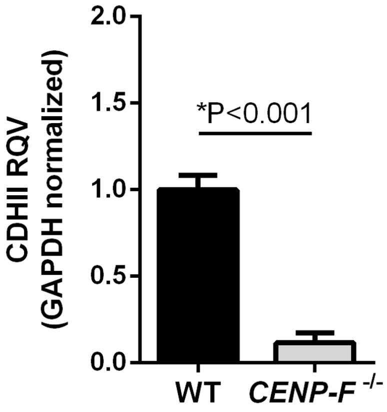

b

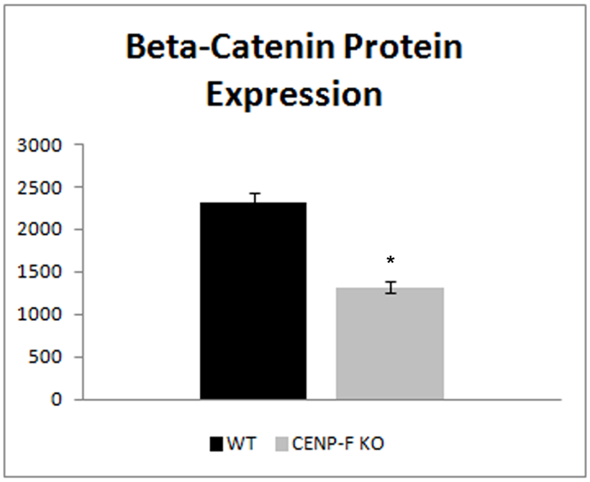

Supplemental Figure 2. Gene expression changes of the cardiomyocyte junctional proteins with loss of CENP-F. The level of n-cadherin is significantly decreased in C*ENP-F*$^{-/-}$ cardiomyocytes when compared to wild-type **(a)**. A western blot analysis of beta-catenin protein expression is significantly decreased in *CENP-F*$^{-/-}$ vs. wild-type cardiomyocytes **(b)** p<0.05, n=5, error bars = (SEM).

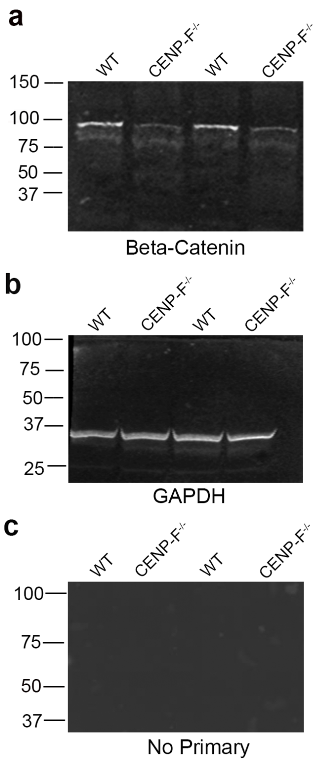

Supplemental Figure 3. Full-length Western Blot of Beta-Catenin shows a significant decrease in *CENP-F*$^{-/-}$ cardiomyocytes. A western blot anaylsis (n=5) shows that loss of CENP-F results in a decrease of beta-catenin expression **(a)**. GAPDH represents a positive control for all western blot analysis **(b)**. No primary represents a negative control for all western blot analysis **(c).**

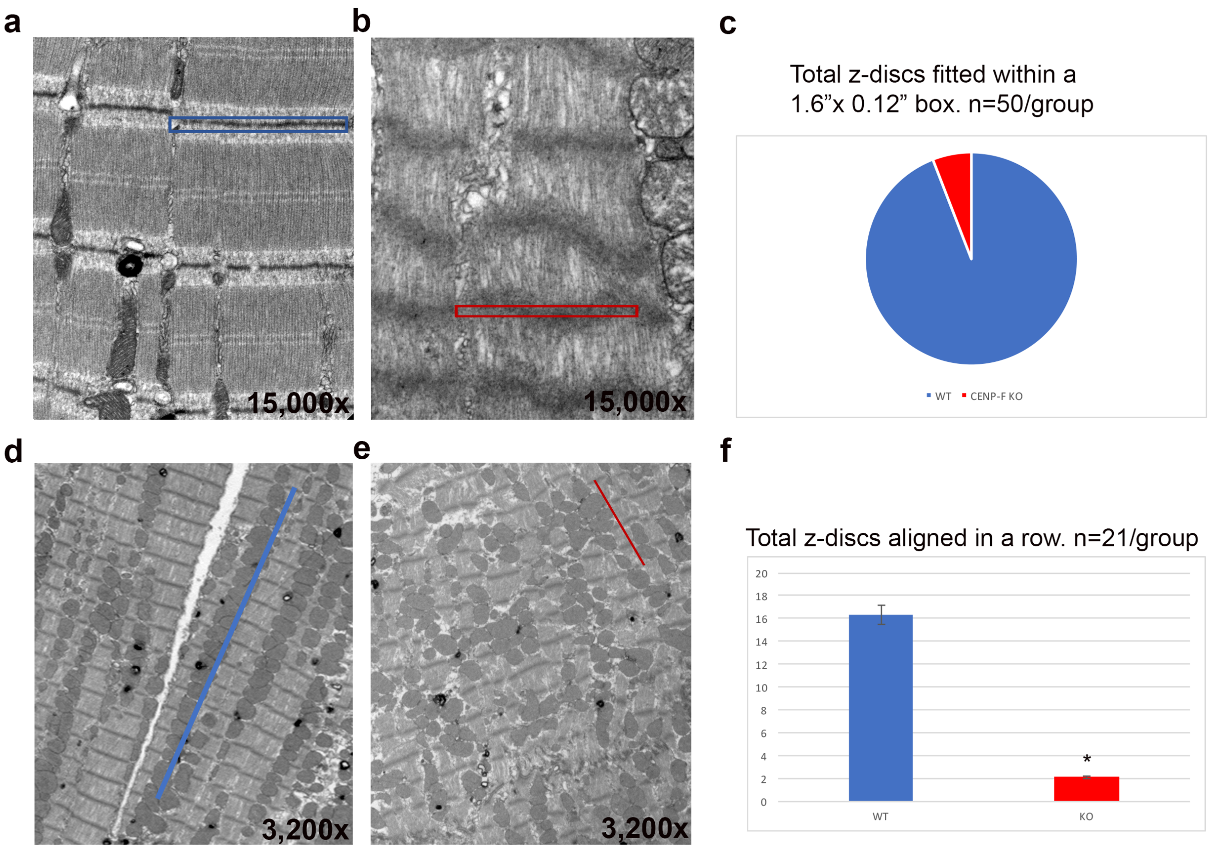

Supplemental Figure 4. Z-discs lose straight and aligned phenotype in *CENP-F*$^{-/-}$ cardiomyocytes. A 1.6" x 0.12" box can fit 96% of wild-type z-discs **(a)** and only 3 out of 50 of the *CENP-F*$^{-/-}$ z-discs could fit **(b)(c)**. As many as 17 z-discs can be counted in a row in wild-type TEM heart images **(d)**. No more than 4 z-discs are aligned in *CENP-F*$^{-/-}$ sarcomeres **(e)(f)**. n=21, *p<0.005. Error bars represent SEM.

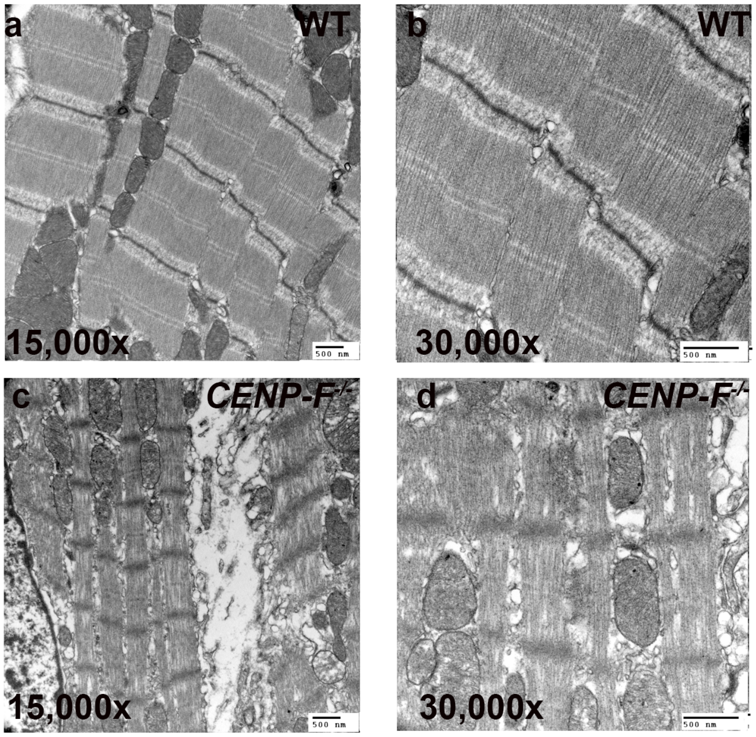

Supplemental Figure 5. The distance between z-discs is shortened in loss of CENP-F hearts. Representative images of sarcomeres in *CENP-F*$^{+/+}$ **(a and b)** and *CENP-F*$^{-/-}$ **(c and d)** hearts demonstrate the shortening of these structures in knockout animals. The sarcomeric distance between z-discs and z-disc thickness was measured in myofibrils with at least four consecutive z-discs **(See Fig. 3 c and d)**. 100 sarcomeres were measured per group from 3 hearts.

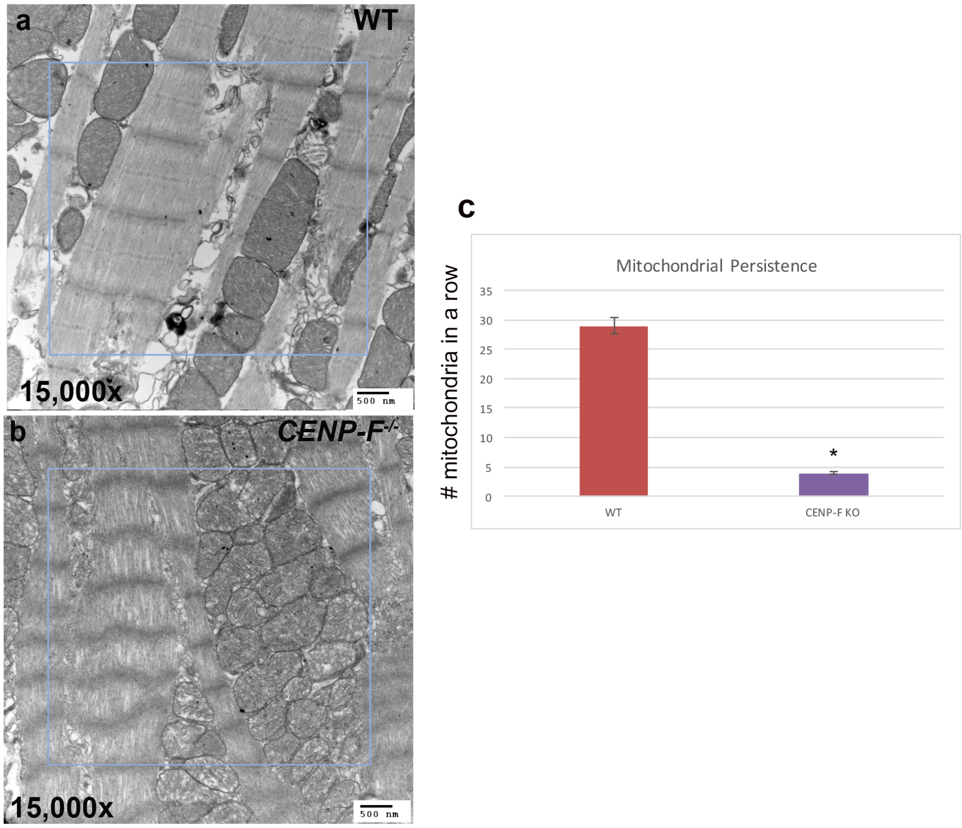

Supplemental Figure 6. Mitochondria are misaligned with loss of CENP-F in TEM images of the heart. In a given area (box), the persistence of mitochondria in wild-type hearts that are aligned within the sarcomeric structures averages at 29 in a row **(a)(c)**. Without CENP-F, there is a significant decrease in mitochondrial alignment averaging only 4 in a row **(b)(c).** $^*p<0.001$, Error bars represent (SEM).

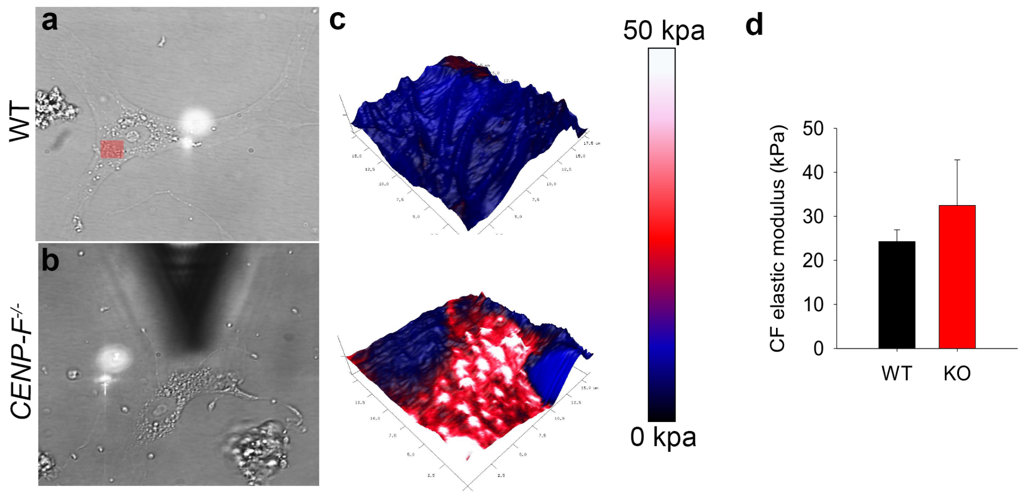

Supplemental Figure 7. Cardiac Fibroblasts are stiffened with the loss of CENP-F. Live cardiac fibroblasts isolated from wild-type **(a)** and *CENP-F*$^{-/-}$ **(b)** mice were plated and analyzed for the elastic modulus of the cell surface. Representative topography plots **(colorbar)** show a soft surface across the 3D rendering of the wild-type cardiac fibroblast **(blue),** while, the *CENP-F*$^{-/-}$ cardiac fibroblast **(red)** is stiffer **(c)**. The average median calculation of the elastic modulus of wild-type vs. *CENP-F*$^{-/-}$ cardiac fibroblasts results in an increase (kpa) **(d)**. Error bars represent (SEM).

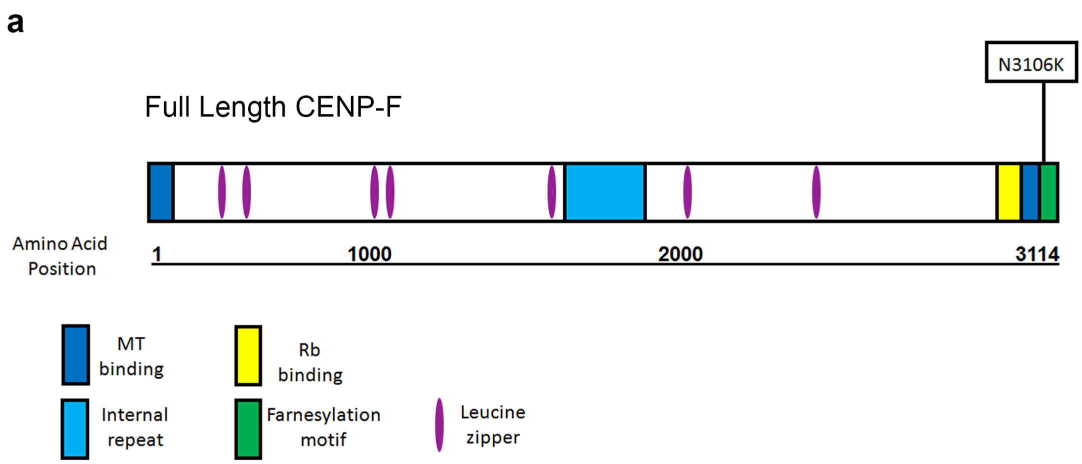

Supplemental Figure 8. CENP-F is relevant in human cardiac disease. In biovu, which includes 25,579 adult DNA samples, a PheWas study identified one common CENP-F variant (rs7289) associated with heart failure with reduced ejection fraction (HFrEF). The CENP-F variant (rs7289) encodes an amino acid substitution, N3106K, at the farnesylation motif (above in green) **(a)**.

Supplemental Table 1

Supplemental Table 1	
Transcript	Amino Acid Change
NM_016343	R300C
NM_016343	H494Q
NM_016343	M701V
NM_016343	Q754E
NM_016343	M793R
NM_016343	R815H
NM_016343	Y1018D
NM_016343	G1033R
NM_016343	T1105I
NM_016343	E1145D
NM_016343	A1208V
NM_016343	L1412S
NM_016343	A1515T
NM_016343	K1539R
NM_016343	N1703S
NM_016343	D1768N
NM_016343	R1879C
NM_016343	D1978G
NM_016343	E2011A
NM_016343	S2044L
NM_016343	A2139T
NM_016343	Q2225R
NM_016343	A2356V
NM_016343	N2396D

NM_016343	R2729Q
NM_016343	N3106K
